# Intrasaccular Flow Disruptor-Assisted Coiling of Intracranial Aneurysms Using the Novel Contour Neurovascular Systems and NEQSTENT: A Single-Center Safety and Feasibility Study

**DOI:** 10.3390/brainsci12080991

**Published:** 2022-07-26

**Authors:** Francesco Diana, Marta de Dios Lascuevas, Simone Peschillo, Eytan Raz, Shinichi Yoshimura, Manuel Requena Ruiz, David Hernández Morales, Alejandro Tomasello

**Affiliations:** 1 Neuroradiology, University Hospital ‘San Giovanni di Dio e Ruggi d’Aragona’, 84125 Salerno, Italy; francesco.diana@gmail.com; 2 Neuroradiology, Vall d’Hebron Hospital Universitari, 08035 Barcelona, Spain; martalascuevas@gmail.com (M.d.D.L.); m.requenaruiz@gmail.com (M.R.R.); dhm050780@hotmail.com (D.H.M.); alejandrotomasello@gmail.com (A.T.); 3 Grupo de Recerca en Ictus, Vall d’Hebron Institut de Recerca, 08035 Barcelona, Spain; 4 UniCamillus International Medical University, 00131 Rome, Italy; 5 Endovascular Neurosurgery, Pia Fondazione Cardinale G Panico Hospital, 73039 Tricase, Italy; 6 Department of Radiology, NYU Langone Health, New York, NY 10016, USA; eytan.raz@gmail.com; 7 Department of Neurosurgery, Hyogo College of Medicine, Nishinomiya 663-8501, Japan; hyogoneuro@yahoo.co.jp

**Keywords:** brain aneurysms, endovascular treatment, coiling, assisted coiling, intrasaccular devices, flow disruptor

## Abstract

*Background:* Intrasaccular flow disruptors (IFD) have been introduced in the treatment of intracranial aneurysms (IAs) to overcome the low aneurysm occlusion rate and the high recanalization rate of the coiling technique. Among them, the Contour Neurovascular System (CNS) and the Neqstent (NQS) were designed to reconstruct the aneurysmal neck and both can be used as assisting coiling devices. We aimed to report our preliminary experience with the flow disruptor-assisted coiling (IFD-AC) technique. *Methods:* We performed a retrospective analysis of prospectively collected data of all patients with IAs treated with the IFD-AC. *Results:* Between February 2021 and April 2022, we treated 15 IAs with the IFD-AC: 10 ruptured and 5 unruptured. The IFD-AC was successfully performed in 13 cases, with a post-operative RROC 1 in 12 cases (92.3%) and RROC 2 in 1 case (7.7%). There was one ischemic event (6.7%) and no hemorrhagic complications. Twelve patients underwent a mid-term radiologic follow-up: Ten IAs (83.4%) presented an adequate occlusion, while 2 (16.7%) had a recurrence. *Conclusions:* The IFD-AC, both with the CNS and the NQS, seems a safe technique with promising efficacy profile. The IFD-AC has proved to be safe without antiplatelet therapy in ruptured cases. Further studies are needed to confirm our preliminary results.

## 1. Introduction

Endovascular treatment (EVT) of intracranial aneurysms (IAs) became the preferred therapeutic modality after the introduction of Guglielmi detachable coils over three decades ago [1,2,3,4,5]. However, coiling of IAs, even with the balloon-assisted technique, raised criticism due to the low post-operative complete occlusion rate, between 57.9% [6] and 76% [7], and the high recanalization rate, which occurs in 8–33.6% of cases [8,9,10,11]. Aneurysm recanalization is associated with bleeding or re-bleeding risks, between 2.7% and 11% [7,12,13], and these risks are proportional to the rate of incomplete occlusion [7].

New devices, called intrasaccular flow disruptors, were developed to overcome these limitations. Nevertheless, randomized controlled trials [14,15,16,17,18] and observational studies [19,20] assessing their efficacy showed rates of adequate occlusion (≈80%) and complete occlusion (≈54%) at the long-term follow-up comparable to coiling.

**Figure 1:**
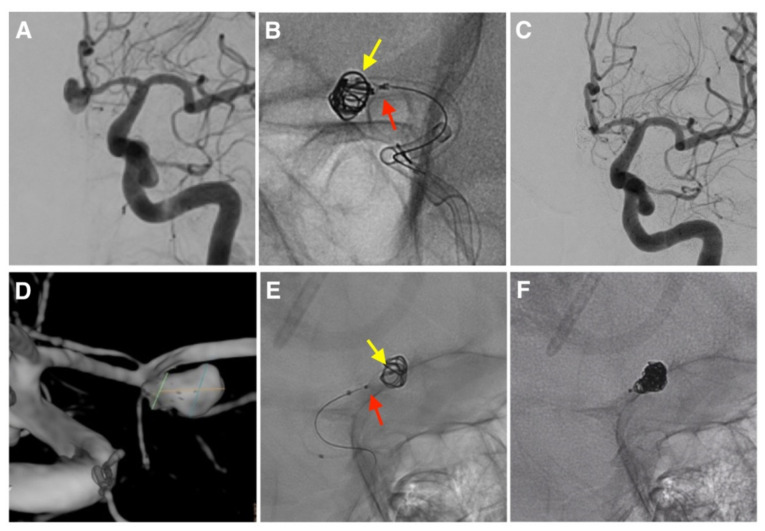
Cases of intrasaccular flow disruptor-assisted coiling (IFD-AC). (**A**–**C**) Case 15 performed with the CNS (red arrow) and a jailed coiling microcatheter (yellow arrow). (**D**–**F**). Case 12 performed with the NQS (red arrow), crossing its meshes with the coiling microcatheter (yellow arrow).

The Contour Neurovascular System (CNS) and the Neqstent (NQS) are intrasaccular devices designed to reconstruct the aneurysmal neck with a dual-layer radiopaque nitinol memory mesh. The NQS, with 48 wires, is the coiling assisting device. The coiling microcatheter can be jailed, or it can cross the meshes. Differently, the CNS, with 144 wires, was conceived as intrasaccular flow disruptor, but it can be used as a coiling assisting device with the jailing technique (Figure 1B). In any case, there are no available data in the literature on the flow disruptor-assisted coiling neither with NQS nor with CNS. This study aimed to report the feasibility and safety of the intrasaccular flow disruptor-assisted coiling (IFD-AC) with the NQS and CNS in the treatment of ruptured and unruptured IAs.

## 2. Materials and Methods

We performed a retrospective analysis of clinical and radiological data prospectively collected in the institutional database of Vall d’Hebron University Hospital of Barcelona. We included all patients with IA treated with the IFD-AC.

We collected baseline patients’ clinical data, admission data, such as modified Rankin Scale (mRS), Hunt and Hess score, and Fisher score, aneurysms’ characteristics, and procedural data (Table 1). We recorded procedural and post-procedural complications, radiological follow-up, done with CT angiography or Digital Subtracted Angiography, and clinical outcomes. The aneurysmal neck coverage of the intrasaccular device was classified as complete or incomplete, if part of the aneurysmal neck was uncovered. The aneurysm occlusion was quantified using the Raymond–Roy Class (RROC) [21]. Clinical outcome was measured with the mRS at the discharge and the 3-month follow-up.

The study was conducted in accordance with the Declaration of Helsinki and approved by the Institutional Ethics Committee of the University Hospital Vall d’Hebron (protocol code PR(AG)564/2021, date of approval 26 November 2021).

### Endovascular Procedure

All cases were performed under general anesthesia, with a trans-femoral or trans-radial artery approach. Patients with unruptured IAs were prepared with dual or single antiplatelet therapy before treatment (Table 1). We did not use antiplatelets in ruptured IAs. We used intraoperative heparin in all cases, administering weight-adjusted doses and monitoring the Activated Clotting Time with a target range of 200–250 s. We routinely infused 15 mg of Nimodipine through the line of the guiding catheter. No other specific intraoperative drug was used.

A 6F long sheath was navigated into the main target artery. A preliminary diagnostic angiography with a three-dimensional rotational angiogram was used for aneurysm sizing and selection of working projections. We followed the sizing algorithm recommended by Cerus Endovascular to select the intrasaccular device. We generally used a bi-axial system for the IFD-AC, except for two cases requiring an intermediate catheter (PhenomPlus 4F—120 cm and SofiaPlus 6F—115 cm). In 10 cases, we used the jailing technique: a 0.017-inch coiling microcatheter (Echelon 10—0.017”ID × 150 cm) was navigated into the aneurysmal sack, subsequently, a NQS or CNS was deployed at the aneurysmal neck through a 0.021-inch microcatheter (Phenom 21—0.021”ID × 160 cm) and detached after coiling (Figure 1A–C). In three cases, we used the coil-through technique. Hence, the NQS was deployed, and its meshes were crossed with the coiling microcatheter (Figure 1E,F).

At the end of the procedure, we performed a control Vaso-CT in all cases.

## 3. Results

Between February 2021 and April 2022, we treated 15 patients with IAs using the IFD-AC: 9 females and 6 males, whose mean age was 61.2 ± 11.6.

Ten patients (67%) presented with aneurysmal subarachnoid hemorrhage (aSAH). At the admission, the median Hunt and Hess score was 3.15 (range 1–5) and the median Fisher score was 4 (range 2–4). In nine of them, the IFD-AC was the first line approach, and it was done within 24/48 h from aneurysm rupture. In one case (Case 6), the IFD-AC was performed after 22 days to retreat an incomplete coiled anterior communicating artery aneurysm (ACoAa).

Five patients with incidental IAs underwent an elective treatment. All of them had a baseline mRS ≤ 2.

Treated IAs had a mean equatorial width of 6.7 ± 2.8 mm, a mean neck width of 3.9 ± 1.5 mm, and a height of 6.9 ± 4.1 mm. Seven of them were located on the ACoA, six on the internal carotid artery (ICA) and two on the basilar tip.

The IFD-AC was successfully performed in 13 cases (Table 2): nine with the NQS and four with the CNS. After deployment of the intrasaccular device, the aneurysmal neck coverage was complete in 11 patients (84.6%) and incomplete in two cases (15.4%).

**Figure 2:**
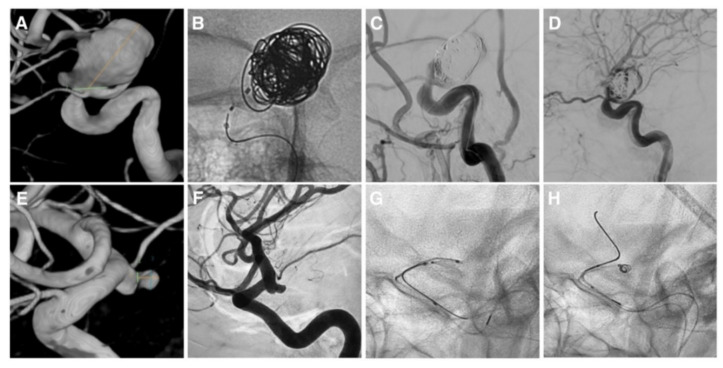
Cases of unsuccessful intrasaccular flow disruptor-assisted coiling. Case 8: (**A**) Supraclinoid ICA aneurysm with severe stenosis of the parent artery. (**B**) IFD-AC performed with the NQS. (**C**,**D**) Flow arrest completely resolved after NQS removal. Case 10: (**E**,**F**) Posterior communicating artery aneurysm. (**G**) Incomplete deployment of the NQS due to the arterial anatomy. (**H**) Treatment continued with a balloon-assisted coiling.

After coiling, we achieved a complete occlusion (RROC 1) in 12 cases (92.3%), a small neck remnant (RROC 2) in one case (7.7%). We failed to perform the IFD–AC in two cases in which we could not detach the device because of anatomical factors. In case 8 (Figure 2A), the NQS detachment was hampered by stenosis of the parent artery. The device was deployed but protruding outside the aneurysmal neck and worsening the parent artery stenosis (Figure 2B). This configuration caused the slowing down of the blood flow to distal territories and the distal embolization of the parieto-occipital M3 branch of the right middle cerebral artery (Figure 2C). The NQS was removed with the consequent arterial flow restoration (Figure 2D). The distal embolism did not cause neurological deficits. In case 10 (Figure 2E,F), the CNS covered the origin of a fetal PCoA (Figure 2G). Consequently, it was removed, and the initial strategy was switched to a BAC without procedural complications (Figure 2H).

Regarding procedural complications, we recorded one (6.7%) minor ischemic event, which did not cause neurological deficits. There were no procedural hemorrhagic complications caused by aneurysm or vessel perforation, no disabling or deathly events.

Among 10 patients with aSAH, two developed significant arterial vasospasm (Case 5 and 8) requiring treatments with intra-arterial injection of Verapamil and mechanical dilatation with stent retriever. Seven patients received external ventricular drainage (EVD), switched to a ventriculoperitoneal shunt (VPS) in one case.

Four patients with SAH had an mRS ≤ 2 at the discharge (40%), while 7 (70%) at the three months follow-up. In-hospital mortality due to SAH complications was 20%. All patients with unruptured aneurysm had an mRS ≤ 2 at the discharge and at the 3-months follow-up.

Radiological follow-up was available in 12 of 13 patients successfully treated with the IFD-AC. Follow-up time ranged between 1 and 215 days. Aneurysm adequate occlusion was seen in 10 patients (83.4%): seven complete occlusions (RROC 1) and three with a small residual neck (RROC 2). Aneurysm recanalization (RROC 3) occurred in two patients (16.7%). Retreatment was done in one patient (Case 1) who underwent a NQS-AC with a final RROC 2. He was retreated with a flow diverter. We had no cases of aneurysm re-rupture.

## 4. Discussion

Intrasaccular flow disruption is a new endovascular approach to treat IAs, aiming to disrupt the intra-aneurysmal flow and to create intra-aneurysmal thrombosis. Intrasaccular flow disruptors have different shapes and sizes. The majority of them occupy the aneurysmal sac, such as the Woven-EndoBridge (MicroVention, Aliso Viejo, CA, USA), the Artisse, and the Medina Embolic Device (Medtronic, Irvine, CA, USA), while the CNS adapts to the lower half of the aneurysm, covering the neck. It is a circular, dual-layered structure of 2 × 72 nitinol wires with one radiopaque platinum marker. It is retrievable and electrolytically detached. It deploys through 0.021” or 0.027” microcatheters. It can also be used as an assisting coiling device, jailing a coiling microcatheter, although it has never been described before. The NQS device is derived from the CNS, made by 48 wires, developed to create an aneurysmal neck scaffolding for coils. This is, to date, the first series reporting results of the new IFD-AC technique, both with the NQS and the CNS.

The feasibility of the IFD-AC is mainly related to the device deployment. In this series, the IFD was successfully deployed in 13 cases (86.7%). This rate is relatively high if compared with the Cerus study [18] in which the CNS was successfully deployed at the first attempt in 21 of 32 patients (66%). Our technical failures were caused by anatomical factors which hamper the usage of the IFD. We can argue that parent artery stenoses, wide-neck aneurysms encompassing the origin of a branch or aneurysms with a small neck unsuited for two parallel microcatheters are the main limitations of the IFD-AC technique.

**Figure 3:**
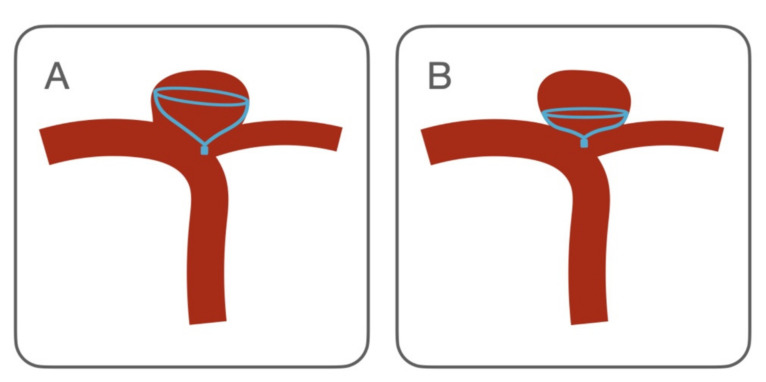
Contour Neurovascular System deployment configurations which may benefit of adjunctive coils. (**A**) CNS incomplete aneurysmal neck coverage. A condition similar to the “dog ear” remnant of the aneurysm clipping, representing a risk for aneurysm recanalization. (**B**) Aneurysmal sack encompassing the origin of an artery. This branch is at risk of being occluded by the CNS. In this case, the CNS is undersized, hence, it opens in a flat configuration, which reduces the anchoring of the device to the aneurysmal wall and increases the risk of intrasaccular migration.

The aneurysmal neck scaffolding created by the IFD is an important factor affecting the long-term outcome [22]. In our experience, the complete neck coverage was achieved 84.6% of cases.The improper positioning or orientation of the IFD creates unfavorable flow conditions [23,24], which affect the immediate and complete aneurysm occlusion. In our opinion the adjunctive coils of the IFD-AC technique can help to face this limitation (Figure 3A). Indeed, just one of the two cases with a residual uncovered neck resulted in a neck remnant after coiling and recurrence at the mid-term follow-up. Moreover, there are other specific cases in which the CNS may benefit from coiling. First, when the CNS is undersized to avoid protrusion in the parent artery. In this case, it has a flat configuration with a reduced grip on the aneurysmal wall. Coils can stabilize the CNS, avoiding its migration inside the sac (Figure 3B). Second, in ruptured cases, to ensure the immediate occlusion of the aneurysm.

We can infer the important role of the IFD-AC in ruptured aneurysms looking at the results of the SAC [25,26]. In ruptured cases the SAC has a high occlusion rate, but the overall complication rate is not negligible (≈20%), and the safety of the proper antiplatelet therapy needed for stenting has not been evaluated in the acute setting. The IFD-AC can keep advantages of the SAC, such as the aneurysmal neck scaffolding that increases coils packaging and trigger the aneurysmal neck endothelialization, and can overcome problems related to the antiplatelet therapy. Indeed, our preliminary experience suggests that the IFD-AC can be performed in ruptured aneurysms without significant embolic complications.

Aneurysm occlusion and recanalization rates are the main concerns of the endovascular treatment [6,12,15,16,27,28]. Among patients treated successfully with the IFD-AC the post-operative aneurysm complete occlusion rate was 92.3%, and the mid-term aneurysm complete occlusion rate was 70%. These are promising results if compared with the results of the Cerus study, in which the immediate complete occlusion rate was 7–10%, and the mid-term complete occlusion rate was 44% [18]. Aneurysm recurrence occurred in two cases, both treated with NQS-AC. There was no recurrence in the group of CNS-AC. Despite the small number of cases, we can speculate that the CNS in assisted coiling may increase the complete occlusion rate, compared to the NQS, due to its structure that allows denser aneurysmal neck scaffolding and higher intrasaccular flow disruption effect. Further studies are needed to confirm this result.

Finally, the safety profile of the NQS and CNS seems acceptable and most likely comparable with other endovascular treatment options.

## 5. Limitations

This study has several limitations. First, the small sample size. The encouraging, clinical and radiological outcomes need to be re-assessed with larger sample sizes. Second, the role of intra-operative antiplatelets, both in ruptured and unruptured cases, need to be assessed in further studies. Third, we included aneurysms with different locations and thus different risk of recurrence [29]. Further studies are needed to assess treatment efficacy in different locations. Fourth, the efficacy of the IFD-AC needs to be assessed in studies with long-term follow-up.

## 6. Conclusions

The intrasaccular flow disruptor-assisted coiling with the Contour Neurovascular System and the Neqstent seems a safe technique for both ruptured and unruptured aneurysms. Our series demonstrated a promising efficacy profile. In ruptured cases, the intrasaccular flow disruptor-assisted coiling has proved to be safe without antiplatelet therapy. Further studies are needed to confirm our preliminary results.

## Figures and Tables

**Table 1:** Summary of baseline characteristics. ACoA: Anterior communicating artery; APT: Antiplatelet therapy; BA: Basilar artery; CNS: Contour Neurovascular System; DAP: Dual antiplatelets; EVD: External ventricular drainage; ICA: Internal carotid artery; OPhA: Ophthalmic artery; PCoA: Posterior communicating artery; SAP: Single antiplatelet.

		1	2	3	4	5	6	7	8	9	10	11	12	13	14	15
**Patients**	Sex	F	M	F	M	F	M	F	F	F	F	M	M	F	M	F
	Age	42	57	75	53	46	51	78	69	73	59	70	77	65	72	50
	1st treatment	Yes	Yes	Yes	Yes	Yes	No	Yes	Yes	Yes	Yes	Yes	Yes	Yes	Yes	Yes
	Baseline mRS	0	5	5	2	0	5	5	5	0	5	0	0	0	0	2
	SAH	Yes	Yes	Yes	Yes	Yes	Yes	Yes	Yes	Yes	Yes	No	No	No	No	No
	Hunt and Hess	2	4	5	2	1	5	5	5	2	3	0	0	0	0	0
	Fisher score	2	4	4	4	4	4	4	4	4	4	0	0	0	0	0
	Treatment timing, day	2	1	1	2	1	22	1	2	2	2	-	-	-	-	-
**Aneurysms**	Location	ICA-OPhA	ACoA	BA	BA	ICA-PCoA	ACoA	ACoA	ICA-supracl	ICA-supracl	ICA-supracl	ACoA	ACoA	ACoA	ICA-supracl	ACoA
	Neck Width, mm	3.5	7.2	4.4	3.5	3.4	5	4.5	6	1.4	3	5	3.3	2.6	3.4	2.4
	Equatorial Width, mm	11	8.5	6.2	7.5	5.7	6	6.4	11	2.7	3.5	6	4	5.2	5.2	4.9
	Height (mm)	17	6.6	8	6	4	7.7	7.4	15	2.7	5.4	5	3.4	3.2	6.8	5
**Devices**	Device	NQS	NQS	CNS	CNS	NQS	NQS	NQS	NQS	NQS	CNS	CNS	NQS	NQS	NQS	CNS
	Size	9	11	7	7	7	7	7	9	7	5	9	7	7	7	5
	APT pre-medication	No	No	No	No	No	No	No	No	No	No	DAP *	SAP *	DAP *	SAP *	SAP *

* DAPT: ASA 100 mg and Clopidogrel 75 mg five days before treatment. SAPT: ASA 100 mg one day before treatment.

**Table 2:** Complications and outcomes. Com: Complete; CTA: CT-angiography; DSA: Digital subtracted angiography; Inc: Incomplete.

		1	2	3	4	5	6	7	8 *	9	10 *	11	12	13	14	15
**Immediate outcome**	Procedure success	Yes	Yes	Yes	Yes	Yes	Yes	Yes	No	Yes	No	Yes	Yes	Yes	Yes	Yes
	Neck coverage	Inc	Inc	Com	Com	Com	Com	Com	-	Com	-	Com	Com	Com	Com	Com
	Intrasaccular flow	No	No	No	No	No	No	No	Yes	No	Yes	No	No	No	No	No
	Final RROC	2	1	1	1	1	1	1	2	1	1	1	1	1	1	1
**Operative-related complications**	Access site	No	No	No	No	No	No	No	No	No	No	No	No	No	No	No
	IA perforation	No	No	No	No	No	No	No	No	No	No	No	No	No	No	No
	Ischemic stroke	No	No	No	No	No	No	No	Yes	No	No	No	No	No	No	No
	Morbidity	No	No	No	No	No	No	No	No	No	No	No	No	No	No	No
	Mortality	No	No	No	No	No	No	No	No	No	No	No	No	No	No	No
**Unrelated complications**	Vasospasm	No	No	No	No	Yes	No	No	Yes	Yes	Yes	No	No	No	No	No
	EVD	No	Yes	Yes	No	No	Yes	Yes	Yes	Yes	Yes	No	No	No	No	No
	VPS	No	No	No	No	No	No	Yes ^	No	No	No	No	No	No	No	No
**Clinical outcomes**	Discharge mRS	0	1	2	1	3	4	4	6	4	6	0	0	0	0	2
	90-days mRS	0	0	0	0	0	2	4	0	5	6	0	0	0	0	0
**Radiological outcomes**	Imaging f-u	DSA	CTA	CTA	DSA	DSA	CTA	-	DSA	CTA	CTA	DSA	CTA	DSA	DSA	CTA
	F-u time, day	180	90	120	215	180	90	-	25	205	3	90	90	180	90	1
	RROC scale	3	2	2	2	1	1	-	1	1	1	1	1	1	3	1
	Retreatment	Yes °	No	No	No	No	No	No	No	No	No	No	No	No	No	No

* Case 8: deployment failed due to stenosis of the parent artery. The device protruded, inducing slowing of the distal flow and subsequent embolism to the distal parieto-occipital M3 segment. The NQS was removed with arterial flow restoration. Case 10: it was a wide-neck aneurysm encompassing the origin of a fetal PCoA. We opted for balloon-assisted coiling to protect the PCoA from CNS and coils protrusion. ^ Case 7: VPS was positioned 72 days after treatment. ° Case 1: the patient was retreated due to aneurysm recurrence with a flow diverter.

## Data Availability

All data relevant to the study are included in the article.
